# From Treatment to Trigger: Steroid-Induced Pancreatitis in the Setting of Leukocytoclastic Vasculitis

**DOI:** 10.7759/cureus.88263

**Published:** 2025-07-18

**Authors:** Yasasvhinie Santharam, Ty J Merry, Alexis M Chrystman, Pramod Reddy

**Affiliations:** 1 Internal Medicine, University of Florida College of Medicine – Jacksonville, Jacksonville, USA

**Keywords:** acute pancreatitis, drug-induced pancreatitis (dip), leukocytoclastic vasculitis, steroid induced pancreatitis, steroid therapy impact

## Abstract

Steroid-induced pancreatitis (SIP) is a relatively uncommon diagnosis, as the main function of glucocorticoids is to decrease cellular inflammation and modulate the immune system. It is a diagnosis of exclusion with a multifactorial pathophysiology and can occur in the setting of treatment with steroids for various underlying conditions. While difficult to diagnose, it can cause patients great discomfort and lead to several complications if missed or treated inappropriately. We seek to highlight a case of SIP in a patient with leukocytoclastic vasculitis who was treated with intravenous and oral steroid therapy, with a clear progression of her pancreatitis seen in serial abdominal imaging.

## Introduction

Acute pancreatitis (AP) is a frequently encountered clinical condition in the hospital, and in the United States, it is the leading cause of hospitalization out of all gastrointestinal disorders [1]. It can be diagnosed using the Revised Atlanta Classification (RAC), which state that two or more of the following thee criteria should be present: 1) abdominal pain suggestive of pancreatitis (acute onset, persistent, severe epigastric pain that sometimes radiates to the back); 2) serum lipase level greater than or equal to three times the upper limit of normal; and 3) characteristic imaging findings on CT scans [2]. The type of pancreatitis can further be classified based on imaging findings. For example, interstitial edematous pancreatitis is seen when pancreatic parenchymal enhancement and/or peripancreatic fluid collections are present. Necrotizing pancreatitis is seen when peripancreatic necrosis is present, there is lack of parenchymal enhancement, or there is an acute necrotic collection of fluid and tissue [2]. The RAC further classifies pancreatitis by severity, rating it as mild (no organ failure, local, or systemic complications), moderately severe (organ failure resolving within 48 hours or local/systemic complications without persistent organ failure), or severe (persistent organ failure >48h) [3].

Some common etiologies of acute pancreatitis are cholelithiasis, alcohol consumption, hypercalcemia, infection, autoimmune disease, and drugs [4]. Over the years, several drugs have been implicated in drug-induced pancreatitis (DIP) [5]. DIP is a multifactorial disease and the pathophysiology is dependent on the specific agent in question. Several theories have been suggested including hypersensitization of the sphincter of Oddi, direct cytotoxic effect, and intrapancreatic activation of pancreatic enzymes [6]. The exact mechanism is drug-specific and often difficult to distinguish. Given that there are so many other causes of pancreatitis, DIP is generally considered a diagnosis of exclusion. 

Several reports of SIP have been well documented in patients with systemic lupus erythematosus (SLE), and steroids were speculated to have increased pancreatic fluid viscosity causing a backup of secretions [7]. However, the pathophysiology appears to be multifactorial and needs further evaluation as multiple components of SLE can lead to an acute pancreatitis episode [8]. Patients without SLE can still suffer from SIP in the setting of other autoimmune diseases requiring steroids such as bullous diseases [9]. We present a case of SIP in the setting of leukocytoclastic vasculitis requiring systemic glucocorticoids.

## Case presentation

**Figure 1:**
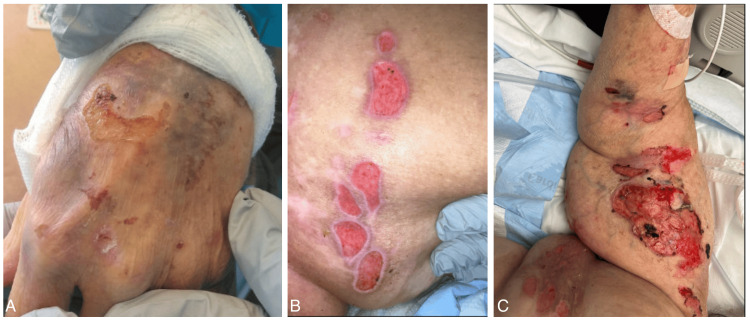
Diffuse erythematous rash with several ruptured bullae A. left hand bullae, B. right thigh ruptured bullae with violaceous borders, C. medial right upper extremity with the majority of the lesions on the patient's inner bicep

A 72-year-old female with a past medical history of hypertension, stage IV chronic kidney disease (CKD), and class II obesity presented to the emergency department for generalized fatigue and a diffuse progressive rash that began approximately two weeks prior. The rash started as a few isolated bullae on the right dorsal forearm that then spread over the rest of the body. The bullae were tense but easily ruptured when manipulated, causing bleeding and intense pain. The patient had no recent exposures to new medications, supplements, or environmental triggers. She was compliant with her lisinopril, metoprolol, and furosemide and had been taking these medications for several months without adverse effects. Physical exam was positive for the diffuse erythematous bullous rash over the bilateral upper and lower extremities, abdomen, torso, back, and left cheek (Figure 1) as well as intertrigo in several of her skin folds. Initial lab testing showed a pre-renal acute kidney injury (AKI) and leukocytosis with 42 x 10^9 cells/L. She was systemic inflammatory response syndrome (SIRS) positive with tachycardia (heart rate >120 beats per minute) and the aforementioned leukocytosis. This prompted an infectious workup including urinalysis, blood cultures, respiratory viral panel, and respiratory culture, which were all negative. She was admitted to medicine for further evaluation of the rash, pain control, and rehydration given her pre-renal AKI on CKD.

**Figure 2:**
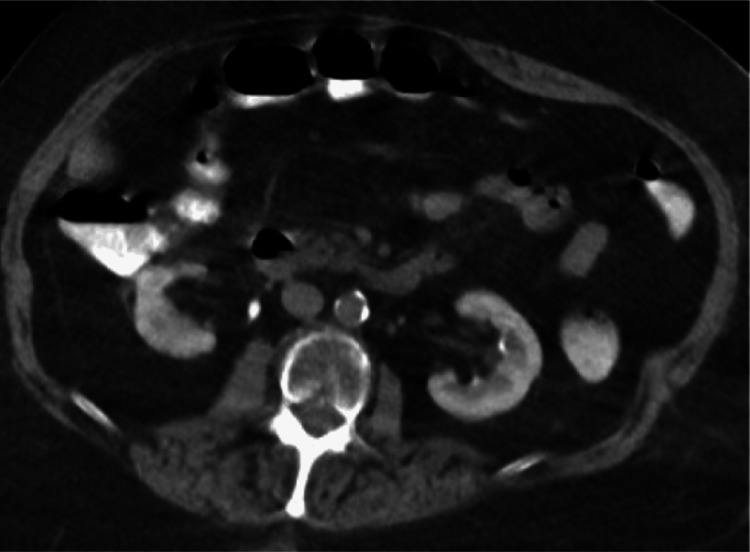
CT scan to rule out malignancy, no abnormalities noted in the abdominal region other than severe cortical thinning of the bilateral kidneys. No pancreatic abnormalities.

Upon examination of the rash, there was concern for bullous pemphigoid versus pemphigus vulgaris. Initial autoimmune testing was negative for antinuclear antibody (ANA), double-stranded DNA antibody (ds-DNA), and rheumatoid factor (RF). On day two of admission, a pan-CT scan was completed to rule out malignancy, with no notable abnormalities in the abdominal region other than severe cortical thinning of the bilateral kidneys (Figure 2). Rheumatology was consulted for further recommendations. Blood tests were run to rule out vasculitis, and were positive for atypical perinuclear anti-neutrophil cytoplasmic antibodies (atypical P-ANCA), though negative for cytoplasmic ANCA (c-ANCA) and p-ANCA. Skin biopsy of a lesion demonstrated positive immunofluorescence for IgG, IgA, IgM, and fibrinoid necrosis compatible with leukocytoclastic vasculitis (LV); immunofluorescence images were not saved at the time. With the positive atypical P-ANCA and skin biopsy showing LV, systemic steroid therapy was started, with plans to begin azathioprine on discharge.

**Figure 3:**
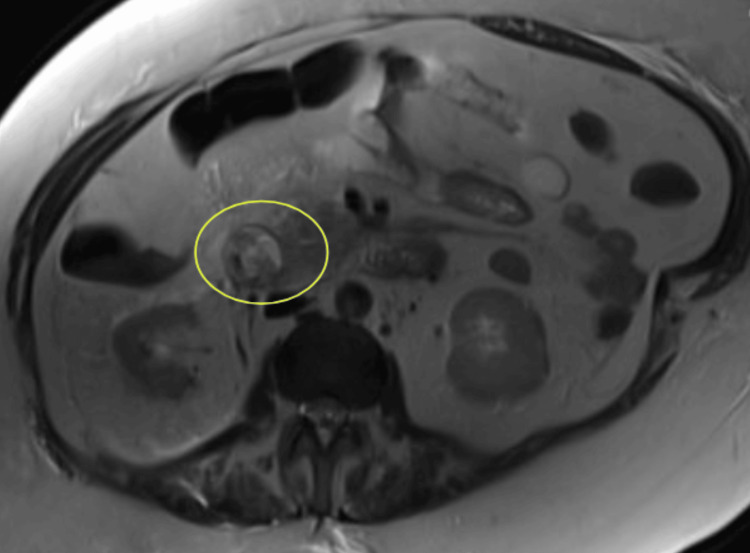
MRI of the abdomen revealing a small amount of fluid (circled in yellow) in the right anterior pararenal space in the center of the pancreatic head.

On days six through 12 of admission, the patient received 125 mg of intravenous methylprednisolone every 24 hours. Of note, during this period on day eight, the patient underwent magnetic resonance imaging (MRI) of her abdomen (Figure 3), revealing a small amount of fluid in the right anterior pararenal space in the center of the pancreatic head. At the time, this was considered a possible chronic finding that may have been missed on the lower resolution of CT, and not further investigated.

**Figure 4:**
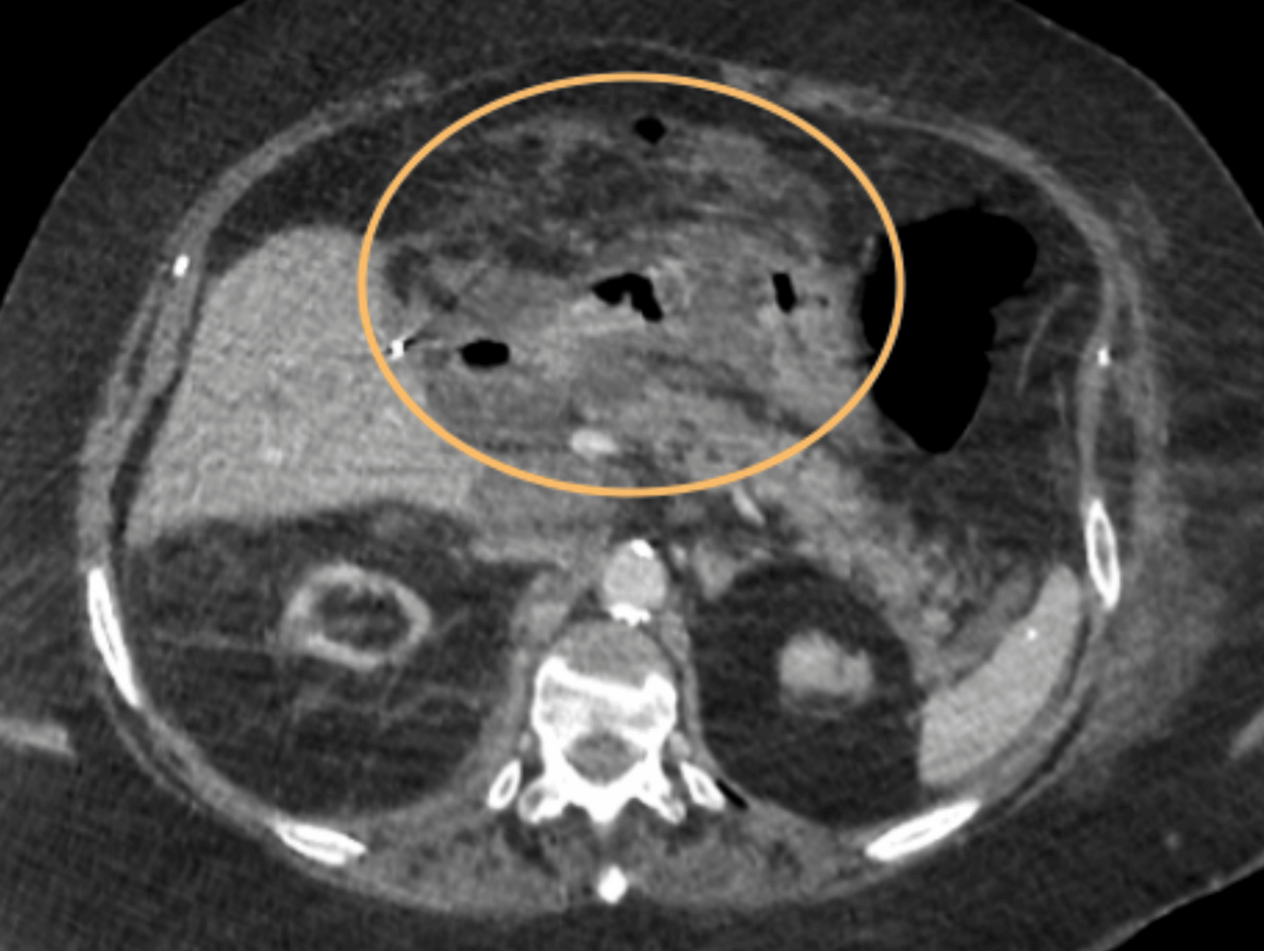
Computed tomography (CT) scan of the abdomen and pelvis with contrast, showing sequelae of acute pancreatitis, with an acute peripancreatic fluid collection in the pancreaticoduodenal groove, measuring approximately 2.3 by 2.9 cm (labeled by the orange circle)

This was transitioned to oral prednisone tablets, of which the patient received 60 mg daily from days 13 to 16, and 40 mg daily on days 17 to 22. On day 22 of admission, the patient suddenly developed sharp epigastric pain, nausea, and vomiting. Lipase was elevated to 841 U/L (>3x upper limit of normal) and a new CT of the abdomen and pelvis with contrast (Figure 4) showed sequelae of acute pancreatitis, with an acute peripancreatic fluid collection in the pancreaticoduodenal groove, measuring approximately 2.3 cm by 2.9 cm. This patient did not have gallstones, biliary dilatation on imaging, hypercalcemia (ionized calcium was 1.12 mmol/L), hypertriglyceridemia (triglycerides were 118 mg/dL), or other medications that could have precipitated the pancreatitis during her hospital stay.

After discussion with rheumatology, it was decided to hold steroids as her bullous rash was improving and steroids may have precipitated the acute pancreatitis. IV fluids and pain medications were begun with improvement of abdominal pain. The patient slowly regained her appetite and transitioned to a regular diet. Repeat CT imaging of the abdomen redemonstrated the fluid collection along with tracking of the fluid in the retroperitoneal space; however, this fluid was unable to be drained by interventional radiology or gastroenterology due to the depth and high-risk location within the abdomen. In the remainder of her hospital course, the patient recovered fully from the acute pancreatitis, with gradual improvement of symptoms over the next four days, and follow-up lipase decreased to 15 U/L. She later spiked a fever with a bandemia. Repeat infectious workup revealed Candida glabrata fungemia in two sets of blood cultures, and micafungin therapy was begun. She was transferred to the Intensive Care Unit for septic shock and acute hypoxic respiratory failure (AHRF). After transfer, the patient vocalized her wishes to be transitioned to comfort care and Do Not Resuscitate/Do Not Intubate (DNR/DNI), passing the following day.

## Discussion

In this patient, other potential causes of AP were ruled out through her history, hospital course, and imaging, before a diagnosis of steroid-induced pancreatitis (SIP) was made. While it is possible that her vasculitis may have contributed to the pancreatitis, the timing did not align given the patient had no abnormalities on abdominal imaging with active vasculitis, the event occurred after several days of steroid therapy whereas it would have improved with steroids if caused by the vasculitis, and resolved swiftly with discontinuation of the offending agent and standard treatment. 

There have been multiple studies that have sought to identify which drugs are associated with drug-induced pancreatitis, with a few classes listed in Table 1 below. In one study, medications were classified based on the number of case reports, the latency (time from medication initiation to development of AP), and a positive rechallenge (re-initiation of the drug causes recurrence of pancreatitis) [10]. In another study specifically investigating the association between steroids and DIP, adverse events reported to the US Food and Drug Administration were analyzed and there was a significant overrepresentation of AP in patients given prednisolone and methylprednisolone compared to other drugs [11]. While studies like this help guide our understanding of DIP, they rely almost solely on case reports, making true causation difficult to establish.

**Table 1:** Steroid-induced pancreatitis (SIP)-associated medications This table has been adapted from Weissman et al. [5], which is an open-access article distributed under the terms and conditions of the Creative Commons Attribution NonCommercial (CC BY-NC 4.0) license.

Medication classes implicated in SIP
Statins
Mesalamine
Certain antibiotics
Steroids
Angiotensin converting enzyme-inhibitors (ACE-i)
Glucagon-like peptide 1 agonists (GLP-1)
Dipeptidyl peptidase IV inhibitors (DPP-4)
Antiviral Medications

Regarding SIP in particular, there are several case reports over the past century as well as a case-control study from Sweden, all of which note the incidence of AP among glucocorticoid recipients [12-14]. The mechanisms behind SIP remain largely unclear. Possible mechanisms include the physiologic alteration of lipid and calcium metabolism that occurs in steroid use, intracellular zymogen release activating intracellular and extracellular enzyme release, and defective intracellular transport leading to activation of intracellular enzymes, with all of these pathways leading to eventual self-digestion of the pancreas and its surrounding tissues [13,15]. 

Another mechanism found after injecting rabbits with steroids hypothesized that corticosteroids might obstruct small pancreatic ductules by leading to increased viscosity of pancreatic secretions, resulting in pancreatic changes. These changes included reduced basophilia, vacuolization of acini, peripancreatic fat necrosis, and hyperplasia of the islets of Langerhans [14].

Management of suspected SIP begins with immediate discontinuation of steroids. This is followed by treatment of AP with early fluid resuscitation using lactated Ringer solution, multimodal analgesia, management of hyperglycemia to avoid secondary pancreatitis infections, and early parenteral nutrition to maintain mucosal integrity of the gastrointestinal tract, with advancement of the diet as tolerated. In this patient, we did see improvement in her AP with the aforementioned measures. Given the full resolution of the pancreatitis prior to the patient’s eventual decompensation, we do not suspect SIP as the etiology of this patient’s mortality.

## Conclusions

Despite their systemic anti-inflammatory and immunosuppressive effects, corticosteroids have been shown to be associated with acute pancreatic inflammation. Steroid-induced pancreatitis is a difficult diagnosis given the multitude of other more common causes of pancreatitis that must be first ruled out. Diagnosis is made through the Revised Atlanta Classification of AP, accompanied by improvement in the patient’s condition with cessation of the offending agent, as well as exclusion of other causes. Management is guided clinically, with treatment of the AP and advancement of the patient’s diet. Here, we see that the development of pancreatitis occurred after the initiation of steroids, which was well visualized on serial CT and MRI abdominal scans. This case highlights the importance of keeping SIP, a relatively rare occurrence, in one’s differential. This will allow for prompt recognition and appropriate management of SIP in order to prevent morbidity and mortality associated with AP.
